# Pressurized Intraperitoneal Aerosol Chemotherapy for Platinum-Resistant Ovarian Cancer: A Systematic Review and Meta-Analysis of Clinical Outcomes

**DOI:** 10.3390/jcm15124443

**Published:** 2026-06-09

**Authors:** Dan Brebu, Flaviu Ionut Faur, Mircea Selaru, Natalia Cireap, Cosmin Burta, Vlad Braicu, Ciprian Duta, Ioana Adelina Faur, Paul Pasca, Amadeus Dobrescu, Georgiana Viorica Moise, Razvan Ilina

**Affiliations:** 1 IInd Surgery Clinic, Timisoara Emergency County Hospital, 300723 Timisoara, Romania; brebu.dan@umft.ro (D.B.); mihai.burta@umft.ro (C.B.); braicu.vlad@umft.ro (V.B.); duta.ciprian@umft.ro (C.D.); adelina.clim@umft.ro (I.A.F.); paul.pasca@umft.ro (P.P.); dobrescu.amadeus@umft.ro (A.D.); 2 X Department of General Surgery, “Victor Babes” University of Medicine and Pharmacy Timisoara, 300041 Timisoara, Romania; selaru.mircea@umft.ro; 3 IIIrd Surgery Clinic, Timisoara Emergency County Hospital, 300723 Timisoara, Romania; 4 Surgical Semiology Department, “Victor Babes” University of Medicine and Pharmacy Timisoara, Eftimie Murgu Square 2, 300041 Timisoara, Romania; razvan.ilina@umft.ro; 5 Surgical Oncology Department, Municipal Hospital, 300172 Timisoara, Romania; 6 Doctoral School of Medicine, “Victor Babes” University of Medicine and Pharmacy Timisoara, Eftimie Murgu Square 2, 300041 Timisoara, Romania; 7 Department of Doctoral Studies, “Victor Babes” University of Medicine and Pharmacy Timisoara, 300041 Timisoara, Romania; georgiana.moise@umft.ro; 8 Medical Oncology, “Pius Brinzeu” Clinical Emergency Hospital, 300723 Timişoara, Romania

**Keywords:** pressurized intraperitoneal aerosol chemotherapy, PIPAC, platinum-resistant ovarian cancer, peritoneal metastases, PRGS, peritoneal cancer index, intraperitoneal chemotherapy, bidirectional therapy, cytoreductive surgery, systematic review, meta-analysis

## Abstract

**Background:** Platinum-resistant ovarian cancer with peritoneal metastases remains a therapeutic frontier marked by limited systemic efficacy and a persistent unmet clinical need for effective locoregional strategies. Pressurized intraperitoneal aerosol chemotherapy (PIPAC) has emerged as a novel minimally invasive platform designed to enhance intraperitoneal drug distribution and overcome biological barriers to chemotherapy delivery. **Methods:** We performed a PRISMA-compliant systematic review and meta-analysis evaluating clinical outcomes of PIPAC in platinum-resistant ovarian cancer. Primary endpoints included histologic regression (PRGS ≤ 2), severe toxicity, and 12-month overall survival, complemented by exploratory analyses of treatment feasibility, disease burden dynamics, and bidirectional therapy strategies. **Results:** PIPAC demonstrated a consistent signal of biologic activity, with pooled histologic response rates indicating meaningful tumor regression despite advanced disease. Severe toxicity remained low across studies, supporting the favorable tolerability of repeated intraperitoneal treatment. Survival outcomes were clinically relevant for a heavily pretreated population, while feasibility analyses suggested that PIPAC may facilitate downstream surgical opportunities in selected patients. Exploratory findings further supported the concept of intraperitoneal disease modulation, reflected by reductions in peritoneal cancer index and integration within multimodal treatment pathways. **Conclusions:** Beyond a purely palliative intervention, PIPAC may represent a biologically active component of personalized treatment strategies for platinum-resistant ovarian cancer. These findings redefine the therapeutic narrative from symptom control toward disease modulation and treatment escalation, underscoring the need for prospective trials to refine patient selection and optimize multimodal sequencing.

## 1. Introduction

Ovarian cancer remains the most lethal gynecologic malignancy worldwide, largely due to its tendency for late-stage diagnosis and diffuse intraperitoneal dissemination [1,2,3,4]. Although advances in cytoreductive surgery, platinum-based chemotherapy, and targeted therapies have improved outcomes in selected patient populations, a substantial proportion of patients ultimately develop platinum-resistant disease [5,6]. Platinum-resistant ovarian cancer (PROC), defined by recurrence within six months following platinum-based chemotherapy, is associated with poor prognosis and limited therapeutic options [7]. In this setting, treatment strategies are predominantly palliative, with modest response rates to systemic therapies and a persistent need for innovative therapeutic approaches capable of addressing intraperitoneal disease [8,9].

Ovarian cancer frequently recurs with diffuse peritoneal dissemination, which remains difficult to control using systemic chemotherapy alone due to limited drug penetration and chemoresistant tumor clones within the peritoneal microenvironment [10,11,12,13,14,15,16,17,18,19,20]. Pressurized intraperitoneal aerosol chemotherapy (PIPAC) is a minimally invasive intraperitoneal drug delivery technique developed to improve spatial drug distribution and tissue penetration in peritoneal metastases. Early clinical studies have reported histologic response and acceptable tolerability in heavily pretreated ovarian cancer patients [21,22,23,24,25].

Preclinical studies have demonstrated superior intratumoral drug uptake under aerosolized pressurized conditions compared with traditional intraperitoneal instillation, providing a strong biological rationale for this therapeutic strategy [26]. Early clinical experiences with PIPAC have reported encouraging signals of antitumor activity in patients with advanced peritoneal metastases from various primary malignancies, including ovarian cancer [27]. In heavily pretreated patients with recurrent or platinum-resistant disease, repeated PIPAC cycles have been associated with histologic tumor regression, stabilization of peritoneal disease, and maintenance of functional status [28,29]. Histologic response is commonly assessed using the Peritoneal Regression Grading Score (PRGS), a standardized histopathological scoring system that allows objective evaluation of intraperitoneal treatment response [30]. In addition to histologic regression, several clinical cohorts have reported reductions in peritoneal tumor burden as measured by the Peritoneal Cancer Index (PCI), suggesting that PIPAC may exert a measurable disease-modifying effect rather than serving solely as a palliative intervention [16,31,32].

Bidirectional treatment strategies combining systemic chemotherapy with intraperitoneal therapy have also been explored in ovarian cancer; however, their relative contribution compared with PIPAC-only approaches remains unclear [33]. In ovarian cancer, where peritoneal dissemination predominates, the potential synergy between systemic therapy and PIPAC represents an attractive therapeutic paradigm. However, the relative contribution of bidirectional therapy compared with PIPAC-only strategies remains poorly defined and has not been systematically evaluated across available studies [34,35,36,37]. Despite increasing clinical adoption and growing interest in PIPAC as a therapeutic option for PROC, the current body of evidence remains fragmented and heterogeneous [38,39,40,41,42,43,44,45,46]. Most published data originate from small prospective trials or retrospective cohort studies conducted in specialized centers, with considerable variability in patient selection, treatment protocols, systemic therapy integration, and reporting of clinical endpoints [47]. Furthermore, previous analyses have focused primarily on survival outcomes and treatment-related toxicity, while other clinically relevant parameters—including treatment feasibility, intraperitoneal disease dynamics, and the potential role of multimodal therapeutic integration—have received comparatively limited attention [48].

A comprehensive synthesis of the available evidence is therefore necessary to clarify the clinical impact of PIPAC in platinum-resistant ovarian cancer [49]. In particular, integrating conventional oncologic outcomes with extended clinical endpoints may provide a more nuanced understanding of how PIPAC fits within the broader therapeutic landscape of recurrent ovarian cancer [50]. The present study was designed to address this knowledge gap through a PRISMA-compliant systematic review and meta-analysis evaluating the efficacy, safety, and clinical impact of PIPAC in patients with platinum-resistant ovarian cancer with peritoneal metastases. In addition to pooled analyses of histologic response, survival, and toxicity, this study incorporates extended clinical parameters including treatment feasibility, peritoneal tumor burden dynamics, and treatment strategy heterogeneity. By integrating these multidimensional outcomes, the current analysis aims to provide a comprehensive evidence-based framework for the role of PIPAC within multimodal treatment strategies for platinum-resistant ovarian cancer [51,52].

## 2. Materials and Methods

### 2.1. Study Design and Reporting Standards

This systematic review and meta-analysis was conducted in accordance with the Preferred Reporting Items for Systematic Reviews and Meta-Analyses (PRISMA) guidelines. The study protocol followed established methodological recommendations for meta-analyses of non-randomized interventional studies in surgical oncology. This systematic review was not prospectively registered in the International Prospective Register of Systematic Reviews (PROSPERO). Although protocol registration is encouraged to enhance methodological transparency and reduce the risk of reporting bias, all stages of the review, including study selection, data extraction, quality assessment, and synthesis of findings, were conducted according to a predefined methodology and in accordance with the Preferred Reporting Items for Systematic Reviews and Meta-Analyses (PRISMA) guidelines.

### 2.2. Search Strategy

A systematic literature search was performed across major electronic databases, including PubMed/MEDLINE, Scopus, and Web of Science, to identify studies evaluating pressurized intraperitoneal aerosol chemotherapy in ovarian cancer. The search strategy combined controlled vocabulary and free-text terms related to “PIPAC”, “pressurized intraperitoneal aerosol chemotherapy”, “ovarian cancer”, “platinum-resistant”, and “peritoneal metastases”. Additional studies were identified through manual screening of reference lists of relevant publications.

### 2.3. Eligibility Criteria

Studies were included if they met the following criteria:

- patients with platinum-resistant ovarian cancer and peritoneal metastases;
- treatment with PIPAC, either as monotherapy or in combination with systemic chemotherapy;
- reporting of at least one clinical outcome of interest, including histologic response (PRGS), survival outcomes, toxicity, or feasibility metrics;
- prospective or retrospective clinical cohort design.

Exclusion criteria included case reports, narrative reviews, conference abstracts without full data, studies lacking ovarian cancer–specific outcomes, or duplicate patient cohorts.

### 2.4. Data Extraction

Two reviewers (F.F.I. and F.I.A.) independently extracted study-level data using a standardized extraction sheet. Variables collected included study design, patient characteristics, treatment regimen, number of PIPAC cycles, and clinical outcomes such as PRGS response, overall survival, progression-free survival, grade ≥ 3 adverse events (CTCAE), non-access rate, conversion to cytoreductive surgery, and PCI dynamics when available. Extended clinical endpoints such as quality-of-life outcomes and treatment feasibility metrics were also recorded.

### 2.5. Outcomes and Definitions

The primary efficacy outcome was the pooled proportion of major or complete histologic regression defined as PRGS ≤ 2. Secondary outcomes included:

- 12-month overall survival;
- grade ≥ 3 treatment-related toxicity;
- procedural feasibility metrics (non-access rate and conversion to CRS/HIPEC);
- exploratory analyses of PCI change and treatment strategy (PIPAC-only versus bidirectional therapy).

### 2.6. Risk of Bias Assessment

Risk of bias was assessed using the ROBINS-I tool for non-randomized studies of interventions. Domains evaluated included confounding, participant selection, classification of interventions, missing data, and outcome measurement. Overall risk of bias was categorized as low, moderate, or serious.

### 2.7. Statistical Analysis

Statistical analyses were performed using R statistical software (R Foundation for Statistical Computing, Vienna, Austria; version 4.4.1). Meta-analyses of proportions were conducted using the packages *meta* (version 7.0-0) and *metafor* (version 4.6-0). Forest plots, subgroup analyses, leave-one-out sensitivity analyses, and exploratory graphical visualizations were generated within the R environment. Statistical significance was defined as a two-sided *p*-value < 0.05. Proportions were pooled using logit-transformed estimates with back-transformation to obtain summary proportions with 95% confidence intervals. Forest plots were generated for PRGS ≤ 2, grade ≥ 3 toxicity, and 12-month overall survival. Subgroup analyses were conducted comparing PIPAC-only and bidirectional treatment strategies, and subgroup differences were evaluated using Q_between statistics. Sensitivity analyses included leave-one-out procedures to assess the robustness of pooled estimates. Exploratory analyses included bubble plots assessing treatment strategy versus histologic response and descriptive visualization of PCI dynamics and feasibility outcomes. Given the limited number of studies, subgroup findings were considered hypothesis-generating.

## 3. Results

**Figure 1:**
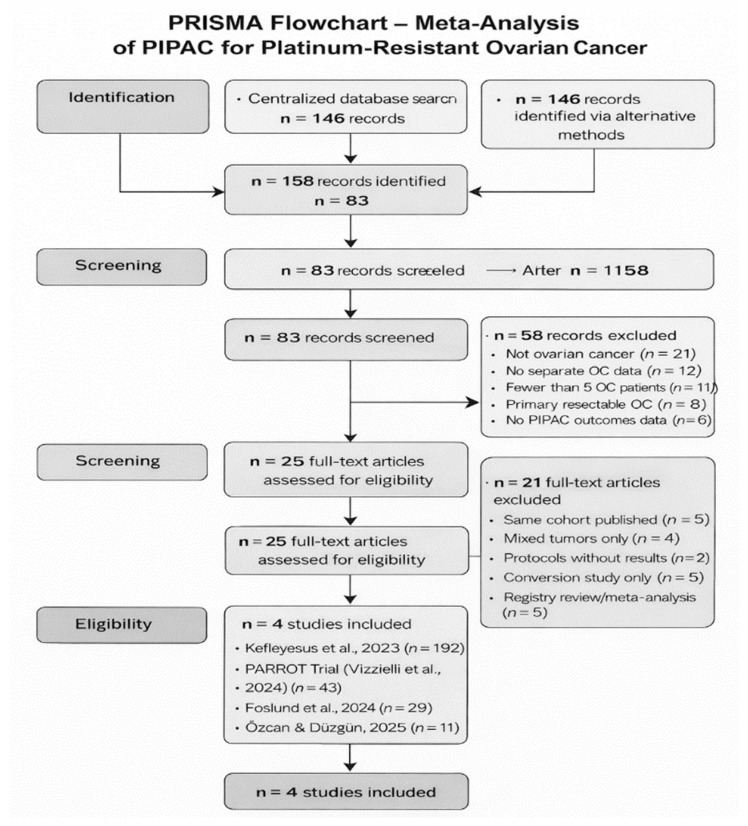
PRISMA Flowchart—Meta-Analysis [20,22,49,52].

The systematic search yielded 158 records, of which 83 unique studies remained after duplicate removal and were screened by title and abstract. A total of 58 records were excluded at this stage due to non-ovarian primary tumors, absence of ovarian-specific data, or lack of relevant PIPAC outcomes. Twenty-five full-text articles were subsequently assessed for eligibility. After rigorous evaluation, 21 studies were excluded, primarily because of overlapping cohorts, mixed tumor populations without extractable ovarian cancer data, protocol-only publications without clinical outcomes, or studies focusing exclusively on conversion surgery rather than PIPAC efficacy. Ultimately, four studies fulfilled the predefined inclusion criteria and were incorporated into the qualitative and quantitative synthesis. The study selection process is illustrated in the PRISMA flow diagram (Table 1 and Figure 1).

Study design, patient population, treatment characteristics, and primary reported outcomes are summarized. Platinum status refers to platinum-resistant (PR) or platinum-sensitive (PS) disease according to the definitions used in each original study. ITT indicates intention-to-treat population, while PP denotes per-protocol analysis. PIPAC regimens predominantly consisted of cisplatin and doxorubicin (C/D). Bidirectional therapy refers to concomitant systemic chemotherapy administered alongside PIPAC.

Histologic response was assessed using the Peritoneal Regression Grading Score (PRGS), with PRGS ≤ 2 representing major or complete histologic response. Survival outcomes include median overall survival (OS) and progression-free survival (PFS) or time-to-progression (TTP) when available. Treatment safety is reported as grade ≥ 3 adverse events according to the Common Terminology Criteria for Adverse Events (CTCAE). Conversion to cytoreductive surgery (CRS) with or without hyperthermic intraperitoneal chemotherapy (HIPEC) and non-access rates at initial PIPAC exploration are also presented when reported. NR indicates outcomes not reported or insufficiently detailed in the original study (Table 2).

Domains evaluated included bias due to confounding, participant selection, classification of interventions, missing data, and outcome measurement. Overall risk of bias judgments were categorized as low, moderate, serious, or critical based on methodological limitations reported in the original publications. Differences in concomitant systemic therapy, retrospective study design, and small cohort size were the main contributors to increased risk of bias across studies (Table 3).

**Figure 2:**
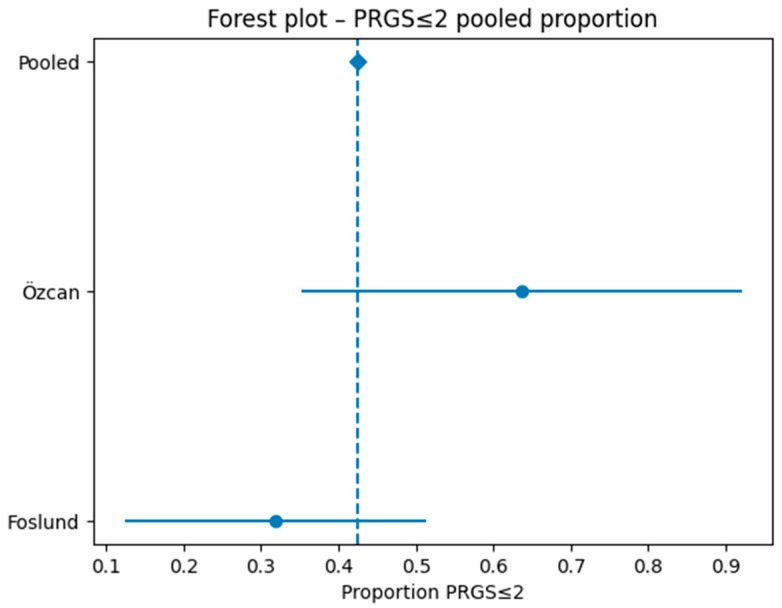
Forest plot—PRGS ≤ 2 pooled proportion.

The pooled analysis showed a PRGS ≤ 2 proportion of approximately 40% across included studies. Individual study estimates varied, with wider confidence intervals observed in smaller retrospective cohorts. Considerable heterogeneity was present across studies regarding patient selection, concomitant systemic therapy, and treatment protocols (Figure 2).

**Figure 3:**
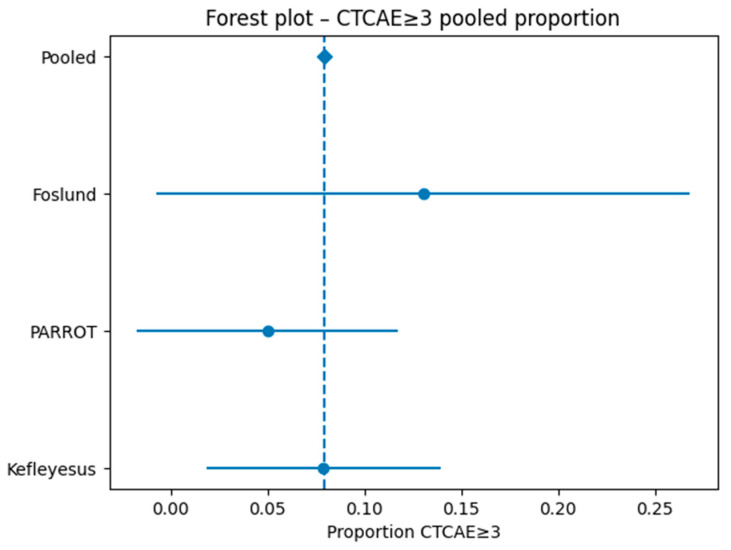
Forest plot—CTCAE ≥ 3 pooled proportion.

The pooled proportion of grade ≥ 3 adverse events remained below 10% across studies, with overlapping confidence intervals between cohorts. Severe treatment-related toxicity was infrequently reported in both prospective and retrospective studies (Figure 3).

**Figure 4:**
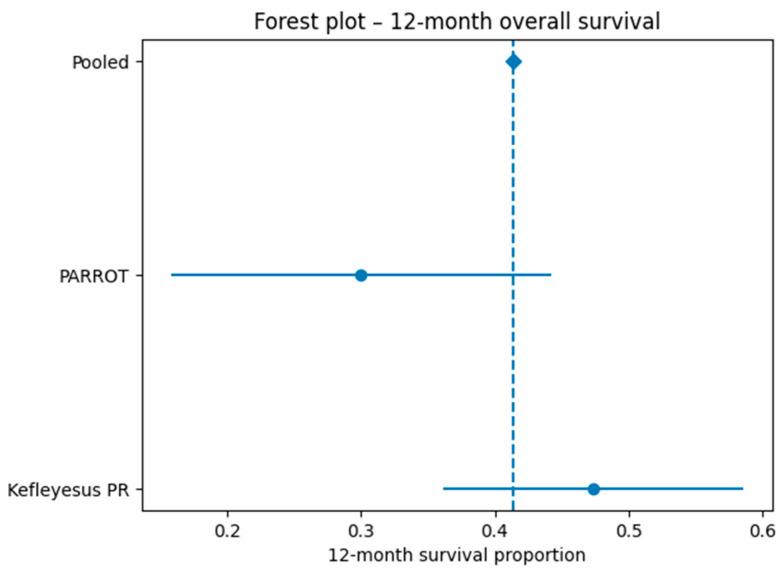
Forest plot—12-month overall survival.

The pooled 12-month overall survival proportion was approximately 40%, although survival estimates varied across studies. Differences in baseline disease burden, prior treatment exposure, and concomitant systemic therapy may have contributed to the observed variability (Figure 4).

**Figure 5:**
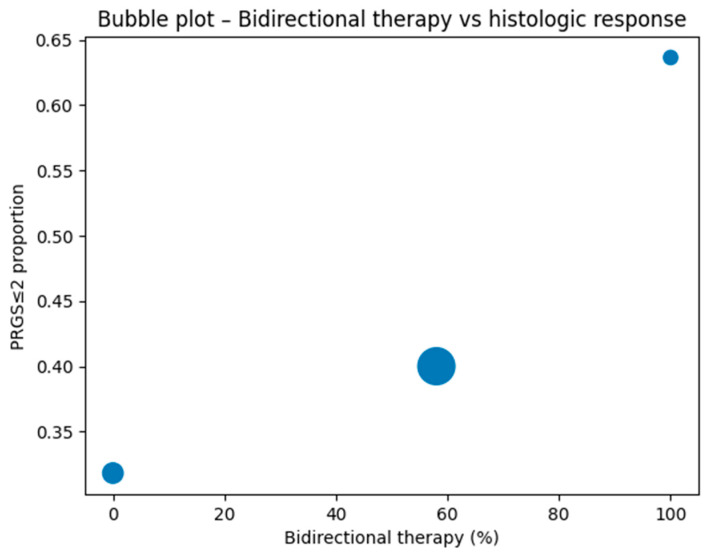
Bubble plot—Bidirectional therapy vs. histological response.

Exploratory analyses showed numerically higher PRGS ≤ 2 proportions in studies with greater use of bidirectional therapy; however, these findings should be interpreted cautiously due to the limited number of included studies (Figure 5).

**Figure 6:**
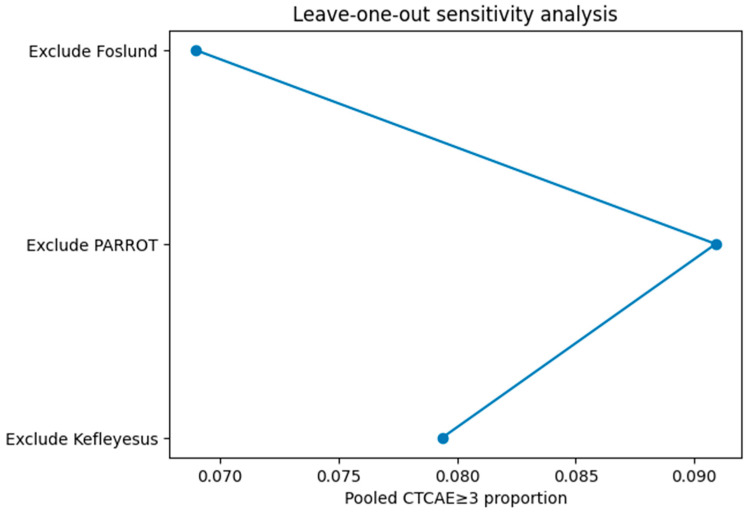
Leave-one-out sensitivity analysis.

Leave-one-out sensitivity analyses showed relatively stable pooled estimates across iterations, without substantial influence from any single study (Figure 6).

**Figure 7:**
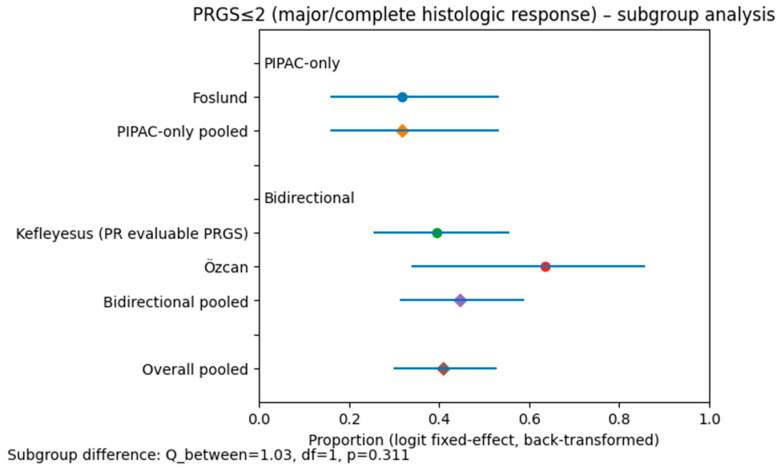
Subgroup forest plot showing the pooled proportion of major or complete histologic response (PRGS ≤ 2) following pressurized intraperitoneal aerosol chemotherapy (PIPAC) in platinum-resistant ovarian cancer, stratified according to treatment strategy (PIPAC-only vs. bidirectional therapy).

In the subgroup analysis comparing PIPAC-only versus bidirectional treatment strategies, the pooled proportion of major/complete histologic response (PRGS ≤ 2) was numerically higher in the bidirectional subgroup. However, the test for subgroup differences was not statistically significant (Q_between = 1.03, df = 1, *p* = 0.311), indicating that observed variability in histologic response across studies may reflect clinical heterogeneity and small sample sizes rather than a consistent subgroup effect (Figure 7).

In subgroup analyses, pooled PRGS ≤ 2 proportions were numerically higher in studies using bidirectional therapy compared with PIPAC-only strategies. However, subgroup differences were not statistically significant (Q_between = 1.03, *p* = 0.311) (Figure 7).

**Figure 8:**
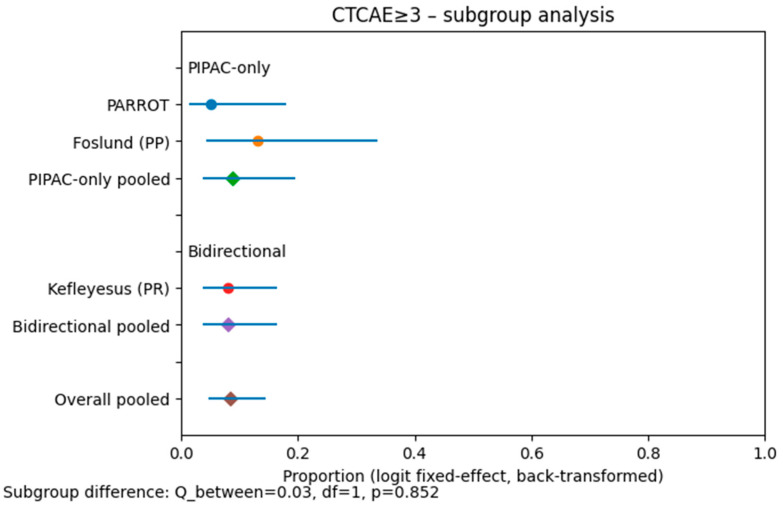
Subgroup forest plot depicting pooled proportions of grade ≥ 3 adverse events according to treatment strategy. Severe toxicity remained low across both PIPAC-only and bidirectional cohorts. Squares represent individual study estimates, and diamonds indicate pooled subgroup and overall estimates. Confidence intervals overlap considerably, and no statistically significant subgroup difference was observed.

Grade ≥ 3 toxicity rates were low across both treatment subgroups, with no statistically significant subgroup difference observed (Q_between = 0.03, *p* = 0.852) (Figure 8).

**Figure 9:**
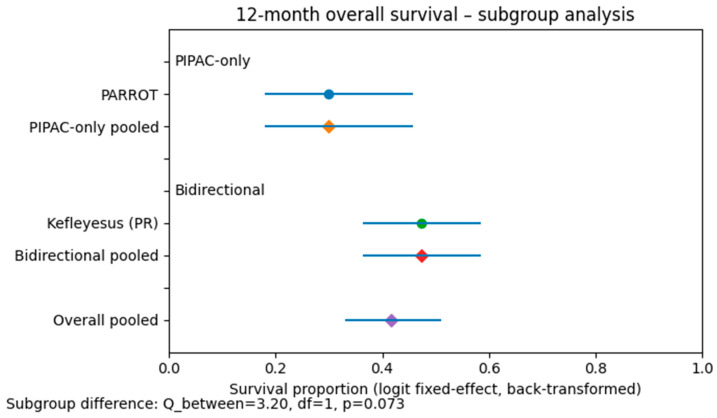
Forest plot showing pooled 12-month overall survival proportions stratified by treatment strategy. Although numerically higher survival was observed in studies with a higher proportion of bidirectional therapy, the subgroup comparison did not reach statistical significance. Estimates were derived from reported survival data and expressed as back-transformed logit proportions with 95% confidence intervals.

The pooled 12-month survival proportion appeared numerically higher in studies with greater use of bidirectional therapy, although subgroup differences did not reach statistical significance (Q_between = 3.20, *p* = 0.073) (Figure 9).

**Figure 10:**
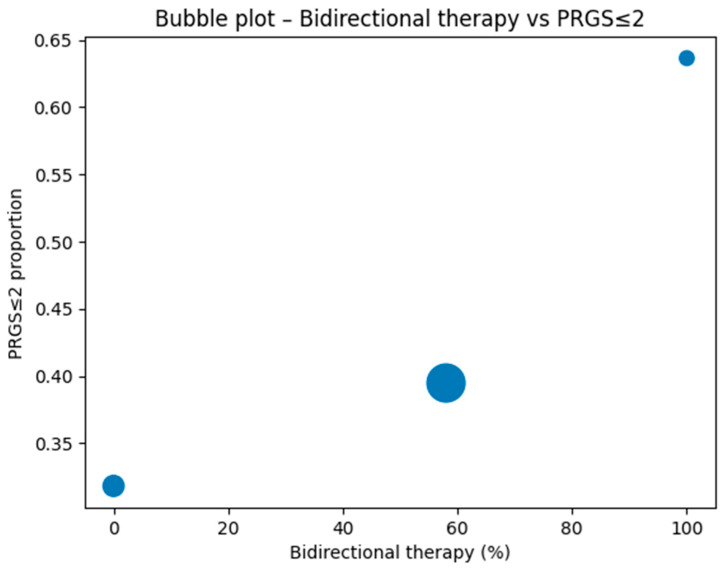
Exploratory bubble plot evaluating the association between the proportion of patients receiving bidirectional therapy and histologic response rates (PRGS ≤ 2). Bubble size reflects study sample size. This analysis is hypothesis-generating and should be interpreted cautiously due to the limited number of included studies and potential confounding factors.

The exploratory bubble plot suggested a trend toward higher PRGS ≤ 2 proportions in studies with a greater proportion of bidirectional therapy. Given the limited number of studies and potential confounding by patient selection and disease burden, this observation should be considered hypothesis-generating (Figure 10).

**Figure 11:**
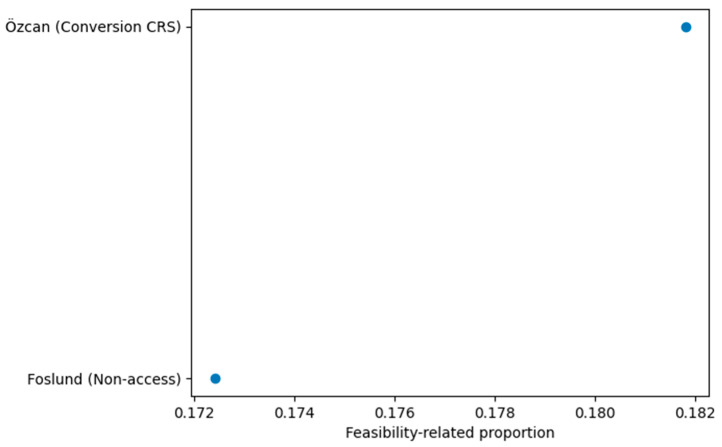
Illustrates key feasibility-related outcomes extracted from included studies. Procedural non-access was reported in approximately 17% of patients in the OPC cohort, whereas conversion to cytoreductive surgery after PIPAC was observed in 18% of patients in the bidirectional cohort. These findings highlight that, despite advanced disease burden, a subset of patients may still achieve surgical conversion, while procedural feasibility remains acceptable across studies.

Conversion to cytoreductive surgery was reported in a minority of patients, indicating that PIPAC may facilitate secondary surgical opportunities in selected cases. Importantly, patient-reported quality of life remained stable in the prospective cohort, supporting the tolerability of PIPAC within a palliative treatment paradigm (Figure 11).

**Figure 12:**
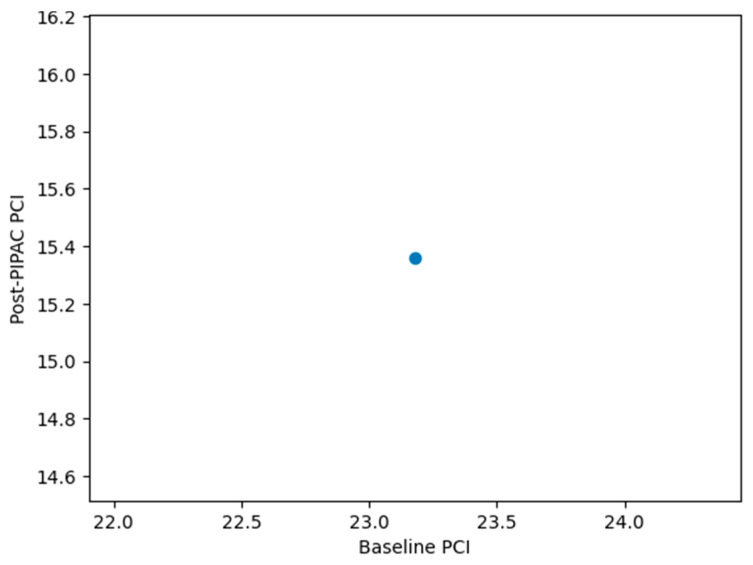
Change in median Peritoneal Cancer Index (PCI) values reported before and after repeated PIPAC cycles in the retrospective cohort described by Özcan et al.

**Figure 13:**
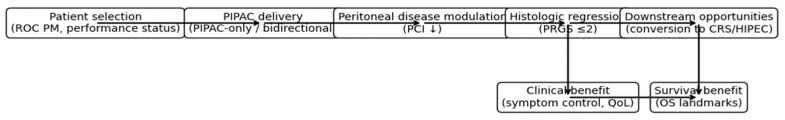
Conceptual overview of the clinical outcomes reported across studies evaluating PIPAC in platinum-resistant ovarian cancer, including histologic response (PRGS), peritoneal disease burden (PCI), feasibility outcomes, quality-of-life measures, and survival-related endpoints.

Across studies, baseline PCI values reflected advanced peritoneal disease, with one cohort reporting a measurable reduction in PCI following repeated PIPAC cycles, suggesting potential disease modulation over time (Figure 12). Treatment intensity varied, with most patients receiving a median of two to three PIPAC cycles, highlighting differences in clinical practice patterns and patient selection. Feasibility outcomes demonstrated acceptable procedural access rates, although a proportion of patients were unable to undergo initial PIPAC due to intra-abdominal factors, emphasizing the importance of patient selection in this setting (Figure 13).

One retrospective cohort reported a reduction in median PCI values following repeated PIPAC cycles. However, this observation was derived from a single uncontrolled study.

Overall, Table 4 provides a broader clinical context for interpreting pooled outcomes by incorporating disease burden dynamics, feasibility parameters, and patient-centered endpoints, thereby strengthening the translational relevance of the meta-analysis.

Overall, the included studies reported histologic response, acceptable tolerability, and feasible repeated administration of PIPAC in selected patients with platinum-resistant ovarian cancer. Exploratory analyses suggested variability according to treatment strategy and patient selection; however, these findings remain limited by the observational nature and heterogeneity of the available evidence. Severe treatment-related toxicity remained low across cohorts, with pooled grade ≥ 3 adverse events below 10%, supporting the favorable safety profile of PIPAC. Importantly, subgroup analyses comparing PIPAC-only strategies with bidirectional treatment approaches revealed no statistically significant differences in toxicity or histologic response, although survival outcomes appeared numerically higher in cohorts with greater use of concomitant systemic therapy. These findings suggest that treatment context and patient selection may contribute to outcome variability more than the delivery technique itself. Beyond conventional oncologic endpoints, extended analyses highlighted clinically relevant feasibility and disease-modulating effects. Procedural non-access occurred in a minority of patients, and conversion to cytoreductive surgery was observed in selected cases, indicating that PIPAC may create opportunities for treatment escalation in otherwise unresectable disease. Additionally, available longitudinal data demonstrated a reduction in peritoneal tumor burden (PCI), supporting the concept of intraperitoneal disease modulation rather than purely symptomatic palliation.

Exploratory bubble plots suggested a potential association between bidirectional therapy and higher histologic response rates, although these findings remain hypothesis-generating due to limited study numbers and heterogeneity. Leave-one-out sensitivity analyses confirmed the robustness of pooled toxicity estimates, indicating that no single study disproportionately influenced the overall results. Taken together, the integrated clinical impact model derived from these analyses suggests that PIPAC may contribute to a multistep therapeutic pathway encompassing peritoneal disease reduction, histologic regression, preservation of quality of life, and potential downstream surgical opportunities, ultimately translating into clinically meaningful survival outcomes in a challenging platinum-resistant population.

## 4. Discussion

The present meta-analysis provides a comprehensive synthesis of the available evidence regarding pressurized intraperitoneal aerosol chemotherapy (PIPAC) in platinum-resistant ovarian cancer (PROC), integrating oncologic efficacy, treatment safety, feasibility metrics, and disease burden dynamics. In a population characterized by limited therapeutic options and predominantly palliative treatment intent, the pooled findings suggest that PIPAC may represent a clinically meaningful adjunct within multimodal treatment strategies. One of the most consistent signals across included studies was the presence of measurable histologic regression, with pooled PRGS ≤ 2 proportions indicating meaningful tumor response despite advanced peritoneal disease. Unlike systemic therapy response metrics, PRGS reflects direct intraperitoneal tumor biology and may therefore provide a more sensitive indicator of local treatment effect. The observation of reduced peritoneal cancer index (PCI) in longitudinal cohorts further supports the hypothesis that PIPAC may induce macroscopic disease modulation rather than merely stabilizing symptoms.

These findings align with emerging concepts suggesting that repeated intraperitoneal drug delivery under pressure may enhance tissue penetration and spatial drug distribution, potentially overcoming the limitations of conventional systemic therapy in platinum-resistant disease. While the heterogeneity of PRGS outcomes likely reflects differences in patient selection, prior treatment exposure, and concurrent therapies, the overall consistency of histologic response supports the biological plausibility of PIPAC in this setting. Pooled survival analyses demonstrated clinically relevant 12-month overall survival rates in a heavily pretreated population, suggesting that PIPAC-based strategies may contribute to prolonged disease control. Subgroup analyses comparing PIPAC-only approaches with bidirectional treatment strategies did not reveal statistically significant differences; however, survival outcomes appeared numerically higher in cohorts with a greater proportion of concomitant systemic therapy.

This observation should be interpreted cautiously, as the treatment strategy likely reflects patient selection rather than a direct causal effect. Nevertheless, the exploratory bubble plot analysis raises the hypothesis that bidirectional approaches may enhance histologic response through synergistic systemic and intraperitoneal drug exposure. Future prospective studies are required to clarify whether combined strategies confer a true survival advantage or simply reflect selection bias toward patients with better performance status. An important finding of this analysis is the consistently low rate of grade ≥ 3 adverse events, reinforcing the favorable safety profile of PIPAC. The minimally invasive laparoscopic approach, combined with reduced systemic exposure, likely contributes to the tolerability observed across studies. Leave-one-out sensitivity analyses confirmed the robustness of pooled toxicity estimates, indicating that the safety signal was not driven by any single cohort.

Beyond safety, feasibility outcomes provide additional insight into the clinical applicability of PIPAC. Procedural non-access occurred in a minority of patients, while conversion to cytoreductive surgery was documented in selected cases. These findings suggest that PIPAC may serve not only as a palliative modality but also as a potential bridge to treatment escalation in carefully selected patients, particularly when intraperitoneal disease burden decreases over time. A key contribution of the present meta-analysis is the integration of extended clinical outcomes beyond traditional survival endpoints. The combined interpretation of PCI dynamics, PRGS response, feasibility metrics, and patient-centered outcomes supports a multidimensional therapeutic framework in which PIPAC may influence several stages of disease management. The proposed integrated clinical impact model illustrates how patient selection, treatment delivery strategy, disease burden modulation, and histologic regression may converge to enable downstream surgical opportunities and sustained symptom control.

Cytoreductive surgery combined with hyperthermic intraperitoneal chemotherapy (CRS/HIPEC) has demonstrated survival benefit in selected ovarian cancer populations, particularly in the interval debulking and platinum-sensitive recurrent settings. However, patients with platinum-resistant disease and extensive unresectable peritoneal metastases are frequently not candidates for aggressive cytoreductive approaches. In this context, PIPAC may represent a less invasive locoregional alternative or complementary strategy, although current evidence remains substantially less mature compared with CRS/HIPEC.

Importantly, quality-of-life data from prospective cohorts indicate that PIPAC does not negatively impact patient-reported outcomes, reinforcing its role within a palliative context. This aspect is particularly relevant in PROC, where therapeutic decisions must balance efficacy with preservation of functional status.

### Limitations

Several limitations should be acknowledged. First, the number of included studies was small, limiting the statistical power and generalizability of pooled analyses. Second, most available data were derived from retrospective or non-randomized cohorts, introducing potential selection bias and confounding. Third, considerable heterogeneity existed regarding patient selection, concomitant systemic therapy, treatment protocols, and outcome reporting across studies. Fourth, several exploratory endpoints, including PCI dynamics and subgroup analyses according to treatment strategy, were based on limited study-level observations and should therefore be interpreted cautiously. Additionally, the small number of studies precluded robust assessment of publication bias and limited the ability to perform formal comparative analyses. Consequently, the present findings should be considered exploratory and hypothesis-generating rather than definitive evidence of clinical benefit. Prospective controlled studies are required to better define the role of PIPAC within multimodal treatment strategies for platinum-resistant ovarian cancer.

## 5. Conclusions

In patients with platinum-resistant ovarian cancer and peritoneal metastases, current evidence suggests that PIPAC may be associated with histologic activity, acceptable tolerability, and procedural feasibility in selected patients with platinum-resistant ovarian cancer and peritoneal metastases. However, available evidence remains limited and predominantly observational, and definitive conclusions regarding survival benefit cannot currently be established. The integration of extended clinical endpoints suggests that PIPAC may contribute to intraperitoneal disease modulation, maintenance of quality of life, and potential downstream surgical opportunities in selected patients. While current evidence remains limited and primarily observational, the present meta-analysis supports the role of PIPAC as a promising component of individualized, multimodal treatment strategies for platinum-resistant disease. Future prospective trials focusing on treatment sequencing, bidirectional therapy integration, and biomarker-driven patient selection are warranted to better define its therapeutic value.

## Figures and Tables

**Table 1:** Characteristics of studies included in the meta-analysis evaluating pressurized intraperitoneal aerosol chemotherapy (PIPAC) in patients with platinum-resistant ovarian cancer and peritoneal metastases.

Study	Design	Population	n (Patients)	Platinum Status	PIPAC Regimen	Concomitant Therapy	Primary Outcomes Reported
**Kefleyesus et al.** [49]	Multicenter retrospective cohort	OC with PM	192 OC analyzed (PR subset included)	PS + PR	PIPAC C/D	Bidirectional common	OS, PFS, PRGS, morbidity
**PARROT Trial (Vizzielli)** [20]	Prospective phase II	Platinum-resistant OC PM	43	PR only	PIPAC C/D	No routine bidirectional	OS, TTP, PRGS, QoL, toxicity
**Foslund et al. (OPC)** [22]	Retrospective subgroup from prospective trials	Advanced OC PM	29 ITT (23 PP)	PR + unresectable	PIPAC C/D	Some systemic	OS, PRGS, AE
**Özcan & Düzgün** [52]	Retrospective single-center	Recurrent PR-OC	11	PR only	PIPAC C/D	Systemic concomitant	OS, PRGS, conversion CRS

Abbreviations: OC = ovarian cancer; PM = peritoneal metastases; PR = platinum-resistant; PS = platinum-sensitive; OS = overall survival; PFS = progression-free survival; TTP = time-to-progression; PRGS = Peritoneal Regression Grading Score; AE = adverse events; CRS = cytoreductive surgery; HIPEC = hyperthermic intraperitoneal chemotherapy.

**Table 2:** Extracted clinical outcomes from studies included in the quantitative synthesis.

Study	PRGS ≤ 2 (Major/Complete)	Median OS (Months)	PFS/TTP (Months)	CTCAE ≥ 3	Conversion CRS/HIPEC	Non-Access
**Kefleyesus**	PR subgroup ≈ 40% (8% CR + 32% major)	11 (PR)	7 (PR)	Severe AE low (reported safe)	NR	NR
**PARROT**	Reported; PRGS improvement significant	27	TTP 12	2/40 (~5%)	NR	NR
**Foslund**	7/22 (32%)	8.2	NR	3 severe events	NR	5/29 (~17%)
**Özcan**	7/11 (63.6%)	15.13	NR	Not fully detailed	2/11 (18.2%)	NR

**Table 3:** Risk of bias assessment of included non-randomized studies according to the ROBINS-I (Risk Of Bias In Non-randomized Studies of Interventions) tool.

Study	Confounding	Selection Bias	Classification of Intervention	Missing Data	Outcome Measurement	Overall RoB
**Kefleyesus**	Serious—bidirectional therapy imbalance	Moderate	Low	Moderate (radiologic missingness)	Moderate	Moderate–Serious
**PARROT**	Moderate (single arm)	Low	Low	Low	Low	Moderate
**Foslund**	Moderate	Moderate (ITT vs. PP)	Low	Moderate	Low	Moderate
**Özcan**	Serious (systemic concomitant)	Serious (n small)	Moderate	Moderate	Moderate	Serious

**Table 4:** Additional feasibility and treatment-related outcomes reported across included studies.

Study	Median PCI (Baseline)	PCI Change	Median No. PIPAC Cycles	≥3 PIPAC Cycles (%)	Non-Access Rate	Conversion to CRS/HIPEC	Ascites/Symptom Control	QoL Outcomes
**Kefleyesus et al.**	Reported (PS vs. PR)	Decrease after repeated PIPAC	Reported	Reported	NR	NR	NR	NR
**PARROT Trial**	Reported	NR	3 cycles median	Reported	NR	NR	Symptom stability described	QoL stable
**Foslund et al. (OPC)**	Reported	NR	Median 2	NR	5/29 (~17%)	NR	NR	NR
**Özcan & Düzgün**	23.18 baseline	↓ to 15.36	Reported	NR	NR	2/11 (18.2%)	NR	NR

## Data Availability

No new data were created or analyzed in this study. Data sharing is not applicable to this article.
